# Evaluation of the effect of *Nigella sativa* oil on the outcome of missed abortion in women: A randomized double‐blind clinical trial

**DOI:** 10.1002/hsr2.2029

**Published:** 2024-04-17

**Authors:** Belgheis Mohammadi, Leila Nazari Robati, Zeinab Tavakol, Mina Movahhed

**Affiliations:** ^1^ Clinical Research Development Unit, Hajar Hospital Shahrekord University of Medical Sciences Shahrekord Iran; ^2^ Department of Medicine, Tehran Branch Islamic Azad University Tehran Iran; ^3^ Community‐Oriented Nursing Midwifery Research Center, Nursing and Midwifery School Shahrekord University of Medical Sciences Shahrekord Iran; ^4^ Department of Traditional Medicine, School of Traditional Medicine Shahid Beheshti University of Medical Sciences Tehran Iran

**Keywords:** misoprostol, missed abortion, *Nigella sativa*, randomized control trial, women

## Abstract

**Background and Aim:**

Due to the concern about the side effects of chemical drugs and their ineffectiveness, the use of natural compounds as alternatives or complementary therapies has received increasing attention. The purpose of this study was to investigate the effect of *Nigella sativa* oil on the outcome of missed abortion.

**Methods:**

In this double‐blind clinical trial, 70 nulliparous pregnant women referred to Hajar Hospital and Imam Ali clinics of Shahrekord and had missed abortion before the 12‐week gestational age were selected and randomly divided into two interventions and control groups. The intervention group received 5 g of *Nigella sativa* oil alone daily for up to 3 days and the control group received a placebo. In case of nonresponse, 3 days after the last dose of medication or placebo, 800 μg of misoprostol (vaginal) were used. Data were analyzed by SPSS software. The chi‐square test, Fisher's exact test, independent *t*‐test and paired *t*‐test were used for analytical statistics.

**Results:**

According to the results, 18 cases (51.4%) in the intervention group and seven cases (20%) in the control group showed complete evacuation of uterine contents which had a significant difference (*p* < 0.05). The frequency of vagina physical examination and type of hemorrhage did not show any significant difference between the two groups before and after the intervention. After the intervention, human chorionic gonadotropin (HCG) was significantly decreased in the intervention group but did not change in the control group (*p* < 0.05). The frequency of adverse events in the intervention group was three (8.6%) and in the control group was one (2.9%) which had no significant difference.

**Conclusion:**

*Nigella sativa* improves the outcome of missed abortion by reducing HCG and facilitating cervix dilatation and delivery of uterine contents.

## 1 INTRODUCTION

Missed abortion means that the products of a stillbirth with a gestational age of less than 20 weeks remain in the uterus for several weeks from the date of the first day of the last period.^1^ It has a prevalence of 6.5–10 cases per 1000 live births, which is equivalent to 10%–20% of pregnancies.^2^ Missed abortion is of particular importance due to serious maternal complications such as coagulation disorders, bleeding, infection, septic shock, uterine rupture, and sometimes maternal death, so it requires medical intervention.^1^

Different methods have been suggested for the management of missed abortion, one of which is expected treatment. The uterus spontaneously excretes most dead fetuses within 3 weeks of the fetus’ death, but the gestational age also affects this process. In addition, the stress and endurance of carrying a dead fetus is painful for the mother even for a few weeks.^3^ Another treatment used for this purpose is surgical treatment. Curettage surgery involves shaving the inner wall of the uterus with a device called a uterine curettage. The most important complication of curettage is uterine perforation, and other complications include cervical rupture, severe bleeding, unsuccessful curettage, infection and remain of products in the uterus following curettage. Frequent curettage to treat a missed abortion also causes adhesions and subsequent infertility.^4^, ^5^, ^6^

In medical treatment, various methods such as progesterone antagonists, high‐dose Syntosynone (Oxytocin) and prostaglandins are used.^3^ One of the most common medical treatments is the use of prostaglandins. Prostaglandins include misoprostol or Cytotec, which is the industrial type of prostaglandin E1. It has uterotonic properties and softens the cervix, which has been used in gynecological diseases, including missed abortion. Low cost, storage capacity and minor effects on the cardiovascular system and bronchial smooth muscle are some of the benefits of this drug. It can also have serious side effects like uterine rupture, coagulation disorders, and severe and abnormal vaginal bleeding.^1^

The effectiveness of misoprostol in the treatment of missed abortion after 48 h is 42%, and the incidence of adverse events in patients treated with misoprostol is significantly higher than that of curettage. However, treatment costs for patients receiving curettage are almost five times higher.^7^ In addition, the cost‐effectiveness of the use of mifepristone and misoprostol has been reported in several studies as being higher than that of misoprostol alone.^8^, ^9^, ^10^

Today, researchers are looking for ways to reduce the side effects of drug treatment for miscarriage or introduce new drugs with fewer side effects. Among these, medicinal plants that have been used by humans for a long time have been considered and the efficiency of some of them such as saffron,^11^ borage,^12^ common rue and marjoram^13^ [7] has been proven.

*Nigella sativa* L. or *Nigella sativa* belongs to the family Ranunculaceae.^14^ Each kilogram of *Nigella sativa* contains 2.5%–0.4% of oil which the main compounds thymoquinone, p‐cymene, carvacrol, dehydro‐sabina ketone, α‐thujene, camphene, α‐pinene, β‐pinene, sabinene, α‐phellandrene, β‐myrcene, γ‐terpinene, limonene, camphor, terpinolene, thymol, carvone, t‐anethol, 4‐terpineol, longicyclene, and sesquiterpene longifolene.^15^, ^16^ The antifertility effect of *Nigella sativa* oil on strengthening fertility has been proven in various studies,^17^ but, due to its strong antiangiogenic activity, its use during pregnancy is prohibited.^18^ Few studies have mentioned the role of *Nigella sativa* in inducing menstruation.^19^, ^20^, ^21^, ^22^ Therefore, there has been no study to evaluate the effectiveness of *Nigella sativa* on abortion and the outcome of missed abortion, whether in animal models or clinical trials.

### 1.1 Objective

This study aims to determine the effect of *Nigella sativa* oil on the consequences of Missed abortion in women who refer to specialized clinics affiliated with Shahrekord University of Medical Sciences.

## 2 METHOD

### 2.1 Study design

**Figure 1:**
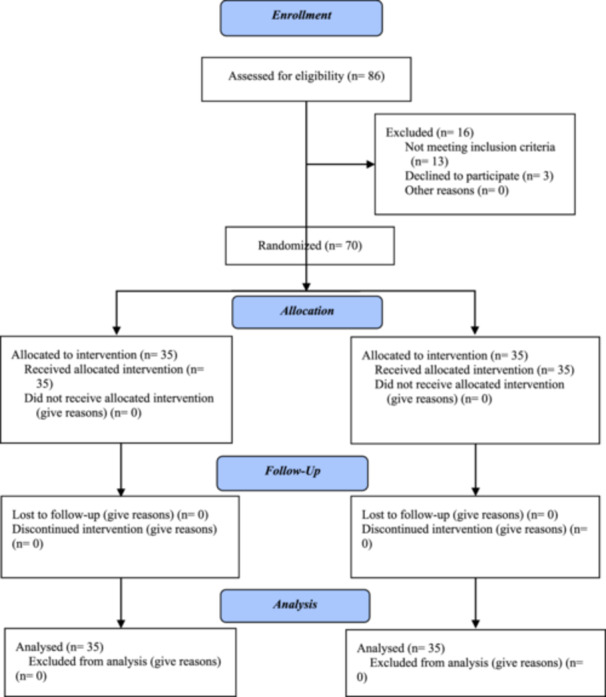
The CONSORT 2010 flow diagram.

This study was performed with the double‐blind control clinical trial method. The study conducted in the Imam Ali Clinic and Midwifery Clinic of Hajar Hospital in Shahrekord. The clinical trial protocol also follows the CONSORT checklist. (Figure 1).

### 2.2 Participants

Study samples were selected among women referring to Imam Ali Clinic and Midwifery Clinic of Hajar Hospital in Shahrekord, from two separate centers based on two separate ultrasound reports indicating the presence of a missed abortion with a gestational age of fewer than 12 weeks.

### 2.3 Inclusion criteria

Inclusion criteria were willingness and satisfaction to participate in the study, neonatal pregnant women with missed abortions with a gestational age of fewer than 12 weeks, lack of specific medical conditions such as diabetes, thyroid dysfunction, kidney disease, etc., ages of 18‐35 years, no history of surgery on the cervix and uterus, normal hematology and coagulation tests, and lack of allergy to misoprostol and *Nigella sativa*.

### 2.4 Exclusion criteria

Patients, who were reluctant to continue with the study at any stage, developed any medical conditions during the study, showed abnormal hematology and coagulation tests, or had evidence of allergy to misoprostol and *Nigella sativa* were excluded from the study.

### 2.5 Interventions

The intervention group was given 5 g of *Nigella sativa* oil daily as an oral capsule for up to 3 days (5 g daily once every 24 h). *Nigella sativa* oil was used for the intervention group 3–4 days before the abortion and a placebo was used for the control group. To make capsules, we used the product of 1 g of *Nigella sativa* oil (made by Barij Essence Factory). Capsules were made of the same shape and size as the placebo, which was coded and produced by the same factory. If the intervention group, after 3 days and receiving 5 g of the *Nigella sativa* oil treatment, showed the dilatation of the cervix and pregnancy products were eliminated, the result was recorded as positive. If the desired result was not achieved, 800 μg of misoprostol were administered vaginally.

For the control group, 800 μg of vaginal misoprostol were given if the abortion was not induced after receiving three doses of placebo. In the absence of response to initial treatment with misoprostol, another dose of 800 μg of vaginal misoprostol suppository was provided for both groups. The name of the misoprostol suppository used in this study was Cytotec, which was made by Searle Pharmaceutical Company in the United Kingdom with a dose of 200 μg per suppository.

### 2.6 Study implementation

A questionnaire that included age, history of miscarriage, diarrhea, vomiting, and fever (before and during medication use) was completed for both groups. In this study, body temperature above 38°C through oral measurement was considered as fever. βhCG levels were determined once before the intervention and once 48 after taking the last dose of medicine. After the intervention, other complications associated with the disease and treatment were investigated. A vaginal examination was performed as soon as vaginal bleeding occurred. After the onset of bleeding and the opening of the cervix, if the content of the uterus had been excreted and there was no need for emergency curettage due to heavy bleeding, ultrasound was performed in both groups to make sure the pregnancy products have been excreted. In case of incomplete excretion of the uterus contents, the clients underwent dilatation and curettage surgery to empty the uterus. In cases of severe bleeding and the need for emergency curettage, the curettage was performed without ultrasound. In this study, by the response to treatment, we mean the opening of the cervix for vaginal examination and complete excretion of pregnancy product with the confirmation of ultrasound.

### 2.7 Outcomes

A missed abortion is a nonviable intrauterine pregnancy that has been retained within the uterus without spontaneous abortion. Typically, no symptoms exist besides amenorrhea, and the patient finds out that the pregnancy stopped developing earlier when a fetal heartbeat is not observed or heard at the appropriate time. An ultrasound usually confirms the diagnosis. No vaginal bleeding, abdominal pain, passage of tissue, or cervical changes are present.^23^ According to the valid midwifery reference books: “Williams Obstetrics, 2014 (Vol. 1, p. 226)” and Beckmann and Ling's Obstetrics and Gynecology (Vol. 8, p. 432), if there are no serious medical disorders in the mother, miscarriage does not require hospitalization.^24^ Therefore, during this study, after obtaining ethical permission from the University's Deputy for Research and obtaining informed consent from the participants, first the blood coagulation factors were checked in all participants and if the blood coagulation factors were normal, the samples were randomly divided into two study groups (using the randomizing software).

### 2.8 Randomization

The selected samples were randomly assigned to the intervention group (*Nigella sativa*) and control group (placebo). To randomize patients into two treatment and placebo groups by a third person and using a computer program with a single randomization method, a sample of random numbers was created based on the volume and a number was allocated to each patient. Even numbers were allocated to the treatment with a semi‐solid topical *Nigella sativa* preparation and odd numbers were assigned to the placebo.

### 2.9 Sample size calculation

The sample size was calculated by using the difference between two independent mean formulas, considering type one error (α) of 05, effect size (d) of 0.72, and power of 80%. The calculation yielded a sample size of 32 for each group. Finally, 35 patients were determined for each group with consideration of a 10% attrition rate.

### 2.10 Statistical analysis

Data were analyzed by using SPSS 16 software. Descriptive statistics were presented as mean, standard deviation, frequency, and percentage. The chi‐square test, Fisher's exact test, independent *t*‐test and paired *t*‐test were used for analytical statistics. The significance level was considered to be 0.05.

### 2.11 Ethical consideration

This study resulted from a doctoral dissertation approved by Shahrekord University of Medical Sciences with a code of ethics IR.SKUMS.REC.1397.205. And it has been registered in the International Clinical Trials Registration Center of Iran with the code IRCT20120709010222N21. In addition, written informed consent was obtained from all participants before the intervention.

## 3 RESULTS

The mean age of the intervention group was 24.74 ± 5.91 years and the control group was 25.57 ± 6.09 years, which did not differ significantly (*p* > 0.05). There was no significant difference between the two groups in terms of other characteristics, including history of uterine and cervical surgery, and drug allergy (*p* > 0.05), (Table 1). Before the intervention, the platelet count, fibrinogen, PTT, and INR levels did not differ significantly between the two groups (*p* < 0.05), but the mean level of PT in the control group was significantly higher than the intervention group (*p* < 0.01, Table 2).

**Table 1:** Demographic characteristics of participants.

Characteristics		Intervention group (No (%))	Control group (No (%))	Test statistic^a^	*p*‐value^a^
Age (mean ± SD)		24.74 ± 5.91	25.57 ± 6.9	−0.578	0.56
History of uterus and cervix surgery	No	28 (80)	27 (77.1)	0.085	>0.99
	Yes	7 (20)	8 (22.9)		
Drug sensitivity	No	35 (100)	33 (94.3)	2.059	0.49
	Yes	0 (0)	2 (5.7)		
History of drug usage	No	31 (88.6)	27 (77.1)	1.609	0.24
	Yes	4 (11.4)	8 (22.9)		

^a^ Fisher Exact Test.

**Table 2:** Comparison of mean and standard deviation of laboratory indexes between two groups.

Laboratory index	Intervention group (mean ± SD)	Control group (mean ± SD)	Test statistic	*p*‐value^a^
Platelets (count)	251.71 ± 47.63	235.29 ± 41.47	1.539	0.13
Fibrinogen (mg/dl)	299.03 ± 21.97	295.91 ± 27.69	0.521	0.60
Prothrombin time (PT)	11.74 ± 0.89	12.47 ± 1.29	−2.756	0.01^a^
Prothrombin time (PTT)	26.25 ± 8.12	29.11 ± 6.67	−1.615	0.11
International ratio (INR)	1.18 ± 0.20	1.26 ± 0.21	−1.618	0.11

^a^ Independent Sample T‐Test.

Based on the results of Table 3, the mean βhCG before and after the intervention in the two groups did not differ significantly (*p* > 0.05). In the intervention group, the mean βhCG decreased significantly after the intervention (*p* < 0.05), but in the nonintervention group, this decrease was not significant (*p* < 0.05). Changes in the βhCG variable during the intervention did not significantly differ between the two groups (*p* > 0.05).

**Table 3:** The mean βhCG before and after the intervention in the two groups.

Level of β‐hCG	Intervention group (mean ± SD)	Control group (mean ± SD)	Test statistic^a^	*p*‐value^a^
Before intervention	7988.57 ± 13309.28	12377.14 ± 25071.17	−0.915	0.36
After intervention	2767.43 ± 5437.65	9249.03 ± 20090.18	−1.842	0.07
**Test statistic** b	2.601	1.099	‐	‐
***p*‐value** b	0.01	0.28	‐	‐
Changes across intervention	5221.14 ± 11873.93	3128.11 ± 16846.40	0.601	0.55

^a^ Independent‐Sample T‐Test.

^b^ Paired‐Samples T Test.

Based on the results of Table 4, the frequency of physical examinations of the vagina and the severity of bleeding in the studied groups before and after the intervention did not differ significantly (*p* < 0.05). The ultrasound showed that 18 subjects (51.4%) in the intervention group had complete excretion of uterine products and in the control group, this occurred in seven people (20%), (Table 4). There was a significant relationship between the results of ultrasound and groups (*p* < 0.05).

**Table 4:** The frequency of physical examinations of the vagina and the severity of bleeding in the studied groups before and after the intervention.

Examination	Time	Result	Intervention group (Proportion (%))	Control group (Proportion (%))	Test statistics	*p*‐value
Vaginal examination	Before intervention	Close	21/35 (60)	23/35 (65.7)	0.392^a^	0.93
		Tip finger	11/35 (31.4)	9/35 (25.7)		
		One Finger	3/35 (8.6)	3/35 (8.6)		
	After intervention	Close	30/35 (85.7)	31/35 (88.6)	1.841^b^	0.55
		Tip finger	3/35 (8.6)	4/35 (11.4)		
		One Finger	2/35 (5.7)	0/35 (0)		
Test statistic***			9.111	10.088	‐	‐
P‐value***			0.23	<0.01	‐	‐
Vaginal bleeding	Before intervention	No bleeding	18/35 (51.4)	17/35 (48.6)	0.448^a^	0.89
		Like menstruation	5/35 (14.3)	7/35 (20)		
		Spotting	12/35 (34.3)	11/35 (31.4)		
	After intervention	No Bleeding	28/35 (80)	22/35 (62.9)	2.934^b^	0.18
		Like Menstruation	0/35 (0)	1/35 (2.9)		
		Spotting	7/35 (20)	12/35 (34.3)		
Test statistic***			2.549	26.429	‐	‐
P‐value***			0.20	<0.001	‐	‐
Result of ultrasound study		Empty	18/35 (51.4)	7/35 (20)	7.529^a^	0.01
		Remains	17/35 (48.6)	28/35 (80)		

^a^ Chi‐square Test.

^b^ Fisher Exact Test.

A comparison of the frequency of side effects in the studied groups is shown in Table 5. According to the results, the frequency of side effects was three people (8.6%) in the intervention group and one person (2.9%) in the control group. There was no significant relationship between the side effects and groups (*p* > 0.05).

**Table 5:** Comparison of frequency of side effects between two groups.

Item		Intervention group (Proportion (%))	Control group (Proportion (%))	Test statistics	*p*‐value^a^
Side effects	No	32/35 (94.1)	34/35 (97.1)	1.061	0.61
	Yes	3/35 (8.6)	1/35 (2.9)		

^a^ chi‐square.

## 4 DISCUSSION

In the present study, which was conducted to determine the effect of *Nigella sativa* oil on the outcome of missed abortion, 70 nulliparous pregnant women with a missed abortion were assigned to two groups of intervention (*Nigella sativa* oil) and control (placebo). According to the results of the present study, the frequency of complete excretion of uterine products in the intervention group (recipient of *Nigella sativa* oil) was 18 people (51.4%) and in the control group was seven people (20%), indicating a significant difference between the two groups. These results indicated an improvement in the excretion of uterine products following the use of *Nigella sativa* oil.

A review of the literature revealed that so far, there has been no study to evaluate the effectiveness of *Nigella sativa* on abortion and the outcome of missed abortion, whether in animal models or clinical trials and in the present study for the first time its effectiveness in improving the outcomes of missed abortion and better excretion of uterine contents was observed. In an ethnobotanical study of the use of herbs to induce abortion, 70% of participants recommended the use of *Nigella sativa* (two doses of 30 g).^13^ In the study of Keshri et al, the contraceptive effects of *Nigella sativa* were evaluated and it was observed that the administration of a hexagonal extract of *Nigella sativa* at a dose of 2 gr/kg daily for 1–10 days after intercourse prevented pregnancy in Sprague Dovli mice.^25^

According to the results of the present study, the mean level of HCG hormone in the intervention group decreased significantly after the intervention compared to before, but this change was not significant in the control group. According to the findings of the present study, the *Nigella sativa* by inducing abortion. How and through which mechanism *Nigella sativa* oil decreases HCG and induces abortion needs further investigation. The results of previous studies have shown that *Nigella sativa* can cause an abortion by inducing menstruation cycles^20^, ^26^, ^27^; and thymoquinone as the main constituent of the essential oil of *Nigella sativa*,^28^ possesses spasmolytic effects by the means of blocking Ca2+ channels, it inhibits the automatic movement of the uterine smooth muscle in guinea pigs and rats and has anti‐oxytocic effects.^29^ In Native Indian medicine, *Nigella sativa* seeds possess emmenagogue effects at doses of 10‐20 mg and high doses can induce abortion.^30^

Given the role of progesterone in maintaining pregnancy, the first step in pregnancy is to reduce progesterone levels. Oxytocin secretion during labor induces uterine contractions, and oxytocin‐induced contractions become stronger and more frequent with progesterone depletion.^4^ Given the above, it is possible that the reduction of progesterone by *Nigella sativa* has caused conditions similar to childbirth and facilitated miscarriage. An experimental study by Ghalandari et al.^31^ on female mice found that treatment with *Nigella sativa* extracts at a dose of 100 mg/kg increased progesterone and reduced it at a dose of 400 mg/kg.

According to the findings of the present study, as well as the above cases, *Nigella sativa* oil may have improved the excretion of uterine contents by affecting hormones such as estrogen, prostaglandins and relaxin. In a study by Abdulrahman et al (2011), the treatment of female mice with *Nigella sativa* oil was associated with a significant increase in serum levels of LH, FSH, progesterone, and estrogen.^32^ Another study by Naseran et al (2020) found that treatment of female mice with *Nigella sativa* and honey significantly reduced LH and significantly increased estrogen and progesterone.^33^ In postmenopausal mice, estrogenic characteristics of *Nigella sativa* were observed through increased serum estrogen.^34^ Thymoquinone is one of the major components of *Nigella sativa*, which has been shown to reduce LH and FSH activity in mice with polycystic ovary syndrome.^35^ Considering the above statements, it seems that the oil of *Nigella sativa* has facilitated uterine content excretion by making changes in the level of hormones involved in childbirth, so it is suggested that in future studies, the effectiveness of *Nigella sativa* on hormone levels in women with missed abortion should also be evaluated.

As mentioned earlier, the widening and softening of the cervix and relaxation of the muscles of the mother's pelvic floor play an important role in the process of childbirth and the excretion of uterine contents. In a study by Aqel et al (1996) in an in vitro environment, the *Nigella sativa* oil had a relaxing effect on the smooth muscles of the uterus of rats and guinea pigs.^20^ In a study by Parvardeh et al (2015) on field mice, the results showed that thymoquinone was able to act as a muscle relaxant and inhibit spasms in skeletal muscle.^36^ Therefore, due to the relaxation effects of *Nigella sativa*, the *Nigella sativa* may have caused the uterine products to exit better by relaxing the cervix.

In the present study, the frequency of side effects was three individuals (8.6%) in the intervention group and one person (2.9%) in the control group, which did not have a significant difference. This result indicates that the use of *Nigella sativa* oils is quite safe. In a study of misoprostol, which is a common treatment for miscarriage, the frequency of side effects such as headache was 26%, uterine cramps was 16%, and diarrhea was 6%, which is higher than the present study.^37^ In a study by Jahangir et al.,^38^ the major side effects of misoprostol included abdominal pain (32%), fever (30%) and vomiting (12.5%).

## 5 CONCLUSION

According to the results of the present study, the use of *Nigella sativa* oil improved the outcome of a missed abortion by improving uterine excretion, so the frequency of complete uterine excretion in the intervention group was 51.4% and in the control group was 20%. In addition, the frequency of side effects in the two groups was not significantly different, which indicates that the use of *Nigella sativa* is safe. Due to its acceptability, efficacy, and minor side effects, it seems that *Nigella sativa* oil can be used to treat missed abortions and its usage in the short term is safe, although further studies with larger sample sizes are needed to confirm its effectiveness as well as its mechanism of action.

## 6 LIMITATIONS

The limitation of this study was the low sample size, so it is suggested to conduct more studies with a larger sample size to confirm the findings.

## AUTHOR CONTRIBUTIONS

**Belgheis Mohammadi**: Conceptualization; Data curation; Investigation; Writing—original draft; Writing—review & editing. **Leila Nazari Robati**: Conceptualization; Investigation; Methodology; Writing—original draft; Writing—review & editing. **Mina Movahhed**: Conceptualization; Investigation; Writing—original draft; Writing—review & editing. **Zeinab Tavakol**: Conceptualization; Data curation; Funding acquisition; Investigation; Project administration; Writing—original draft; Writing—review & editing.

## CONFLICT OF INTEREST STATEMENT

There is no conflict of interest in this study.

## TRANSPARENCY STATEMENT

The lead author Zeinab Tavakol affirms that this manuscript is an honest, accurate, and transparent account of the study being reported; that no important aspects of the study have been omitted; and that any discrepancies from the study as planned (and, if relevant, registered) have been explained.

## Data Availability

All data underlying the results presented are available in the article. The authors confirm that the data supporting the findings of this study are available within the article.
